# Attachment and Concord of Temporal Adverbs: Evidence From Eye Movements

**DOI:** 10.3389/fpsyg.2019.00983

**Published:** 2019-05-31

**Authors:** Nicoletta Biondo, Francesco Vespignani, Brian Dillon

**Affiliations:** ^1^Basque Center on Cognition, Brain and Language (BCBL), San Sebastián, Spain; ^2^Fondazione Marica De Vincenzi ONLUS, Rovereto, Italy; ^3^Department of Psychology and Cognitive Science, University of Trento, Rovereto, Italy; ^4^Department of Linguistics, University of Massachusetts (UMass), Amherst, MA, United States

**Keywords:** tense, temporal adverbs, temporal concord, attachment, eye movements, memory retrieval, sentence comprehension

## Abstract

The present study examined the processing of temporal adverbial phrases such as “last week,” which must agree in temporal features with the verb they modify. We investigated readers’ sensitivity to this feature match or mismatch in two eye-tracking studies. The main aim of this study was to expand the range of concord phenomena which have been investigated in real-time processing in order to understand how linguistic dependencies are formed during sentence comprehension (Felser et al., 2017). Under a cue-based perspective, linguistic dependency formation relies on an associative cue-based retrieval mechanism (Lewis et al., 2006; McElree, 2006), but how such a mechanism is deployed over diverse linguistic dependencies remains a matter of debate. Are all linguistic features candidate cues that guide retrieval? Are all cues given similar weight? Are different cues differently weighted based on the dependency being processed? To address these questions, we implemented a *mismatch paradigm* (Sturt, 2003) adapted for temporal concord dependencies. This paradigm tested whether readers were sensitive to a temporal agreement between a temporal adverb like *last week* and a linearly distant, but structurally accessible verb, as well as a linearly proximate but structurally inaccessible verb. We found clear evidence that readers were sensitive to feature match between the adverb and the linearly distant, structurally accessible verb. We found no clear evidence on whether feature match with the inaccessible verb impacted the processing of a temporal adverb. Our results suggest syntactic positional information plays an important role during the processing of the temporal concord relation.

## Introduction

Sentences are routinely made of words whose formal properties need to covary in order to reach grammaticality. This relation among words, which has been generally called agreement or concord (Corbett, 2003), can involve several elements such as the subject noun phrase and the verb of a sentence (e.g., *The man is washing the car*), and/or the subject noun phrase and an anaphoric pronoun (e.g., *The man is washing himself*). In addition to being pervasive features of human language, concord phenomena have attractive properties for researchers investigating the interplay between memory and sentence comprehension. For example, consider a sentence such as *the pasta recipe from the northern provinces tastes amazing.* Comprehending this sentence requires the reader to integrate the subject phrase, headed by *the pasta recipe*, with the verb *tastes*. Because these terms are not linearly adjacent in the input, this process plausibly requires memory retrieval: the comprehenders must encode the subject noun phrase, and have some mechanism for reactivating or retrieving the information in that encoding when it is needed, at a later point in processing (Lewis et al., 2006; McElree, 2006). This intuition lies at the heart of *cue-based parsing models*, which hypothesize that incremental sentence processing relies on a fast, associative, cue-based retrieval mechanism to reactivate linguistic encodings in memory when those encodings are necessary to parse or interpret the current input (for overviews, see Lewis et al., 2006; McElree, 2006; Foraker and McElree, 2011;Van Dyke and Johns, 2012; Wagers and McElree, 2013).

From this perspective, concord phenomena are useful to study in sentence processing, because the linguistic features marked on one element may provide important *retrieval cues* that can help comprehenders retrieve previously processed linguistic encodings. For example, the agreement morphology on the verb *tastes* in the example above might provide a (SING) feature that could be used to reactivate or retrieve the subject phrase *the pasta recipe* at the verb *tastes*. There are many empirical and theoretical questions raised by this hypothesis. Are all linguistic features that participate in agreement relations used as retrieval cues? Do all potential linguistic constraints belong to the set of cues that guide retrieval? If so, are all linguistic cues given similar weight, or do some specific cues, such as structural cues, have a leading role in the set of available cues used during memory retrieval (Van Dyke and McElree, 2006, 2011; Dillon et al., 2013; Patil et al., 2016; Parker and Phillips, 2017; Kush et al., 2018)? Researchers addressing these questions have largely focused on how comprehenders implement agreement and anaphoric dependencies in online comprehension and how different cues, such as structural cues and morphological cues, are differently weighted during the processing of these dependencies (Felser et al., 2017; see Jäger et al., 2017 for a comprehensive summary and meta-analysis).

In this paper, we try to extend the empirical basis of this literature by investigating a different and less typical concord phenomenon, namely the relationship between a deictic temporal adverb such as *last month* and its match with the temporal information expressed by the verb of the sentence. An example is given in (1):

(1) The postman who **used to work** in Yonville **delivered** a nice gift to me **last month**.

The processing of the adverb-verb temporal concord dependency is a good place to investigate the role of memory retrieval in syntactic processing. The successful attachment of a deictic temporal adverb such as *last month* in (1) would require finding a grammatically accessible verb phrase to modify (a *structural* constraint). Moreover, the adverb must express temporal information that is coherent with the temporal information expressed by the finite verb (a *morphosyntactic* constraint). Both these types of constraints - structural constraints that determine *where* the adverb can attach, and morphosyntactic constraints that determine *what temporal features* that attachment site must have - could plausibly be used as retrieval cues during memory retrieval in a cue-based parsing model. The morphological (i.e., temporal) cue provided by the adverb is triggered/available at the same time in which a structural cue is initiated (in order to find an appropriate structural placement). In addition, it seems very plausible that retrieval processes would be necessary to fully integrate a temporal adverb into an unfolding parse. This is because adverbs constitute optional constituents that cannot be reliably anticipated; the processing of an adverb might therefore not receive much facilitation due to the predictive computation of syntactic structure (e.g., the left-corner parsing framework in Lewis and Vasishth, 2005). It would thus seem that processing of temporal adverbs is ideal to study the interplay between structural and morphological cues during memory retrieval.

In this paper, we will first consider the processes necessary to integrate a temporal adverbial into a sentence. We will then turn to a consideration of how cue-based parsers realize these different processes. We then present two eye-tracking experiments that investigate the role of morphosyntactic and structural constraints on the processing of deictic temporal adverbs.

### Adverb-Verb Attachment and Concord: Previous Studies

Each time a temporal adverb is encountered there are two potentially distinct processes that need to occur in order to reach a complete and coherent temporal interpretation of the event expressed in the sentence. One, a structural attachment site must be found to integrate the adverb into the syntactic structure. This attachment site is provided by the maximal projection of the phrase modified by the adverb (Chomsky, 1986, 1995; Sportiche, 1988 among others), such as the Temporal Phrase (TP) or the Verb Phrase (VP).^1^ Two, a temporal feature match must be established between the deictic temporal adverb^2^ and the tensed verb it modifies, in order to successfully define the temporal location of the event expressed by the verb. Existing experimental evidence suggests that both processes—attachment and concord—occur during the incremental processing of the adverb-verb relation.

Evidence concerning the attachment process of temporal adverbs in incremental comprehension comes from studies on syntactic ambiguity resolution. For example, Altmann et al. (1998) measured the reading times on a temporal adverb such as *next week* in syntactically ambiguous sentences (e.g., Fiona *implemented* the plan she *proposed next week*). Altmann et al. (1998) manipulated the temporal features of these two attachment sites to force the low attachment of the adverb (e.g., Fiona *implemented* the plan she *will propose next week*) or the high attachment of the adverb (e.g., Fiona *will implement* the plan she *proposed next week*). This eye-tracking study showed longer reading times (from early measures on) for the high attachment condition compared to the low attachment condition (see similar results in Van Gompel et al., 2005). In sum, there is evidence that low attachment of the temporal adverb was generally preferred (i.e., more easily processed) than high attachment. The low attachment preference has been related to general recency effects: the parser attaches the adjunct to the most recent and/or active verb phrase (e.g., MacDonald et al., 1994; Gibson et al., 1996). Alternatively, the low attachment preference has also been related to parsing principles such as the *Late Closure* principle (Frazier, 1979), or *Construal* (Frazier and Clifton, 1996) which holds that new phrases (e.g., adverbs) are preferably attached to the current phrase (or thematic domain) being processed.

Evidence that comprehenders evaluate temporal concord between a verb and a temporal adverb in real-time comes primarily from event-related potential (ERP) studies investigating the electrophysiological activity triggered by a grammatical violation during sentence processing. These studies have shown that a violation of the concord relationship between a deictic temporal adverb and the verb tense (e.g., *Yesterday I sailed/^∗^sail*) yields ERP components characteristically associated with both syntactic and semantic anomaly detection. Relative to an acceptable baseline, sentences containing a verb that mismatches in temporal features with a deictic temporal adverb yields negative ERP deflections in early time windows (e.g., 300–500 ms) and positive ERP deflections in later time windows (e.g., 600–900 ms) after the verb onset. This is sometimes characterized as a LAN-P600 complex (Steinhauer and Ullman, 2002; Baggio, 2008), sometimes as an N400-P600 complex (Dillon et al., 2012; Qiu and Zhou, 2012). More recently, it has also been shown that when a temporal mismatch occurs between a deictic temporal adverb and a distal verb (e.g., *Yesterday afternoon the tired traveler ^∗^will come/came back home*) longer reading times are found compared to the correct control condition, both in early and late eye-tracking measures (Biondo et al., 2018).

### Adverb-Verb Attachment and Concord: A Cue-Based Perspective

The evidence briefly reviewed above lends support to the idea that the parser needs to find a structurally appropriate attachment site for an adverb, and that it evaluates temporal concord between an adverb and a verb. Some parsing models treat sentence structure building (e.g., attachment) and the check of feature consistency as two independent and temporally ordered operations, potentially subserved by distinct processing mechanisms (e.g., Frazier, 1987; Friederici, 2002). The distinction between these different processes is less clear-cut in cue-based parsing models (e.g., Lewis and Vasishth, 2005). This is because both syntactic and morphological features can be used as retrieval cues that guide the memory retrieval processes necessary to integrate the adverb into the sentence. In this sense both constraints are “enforced” at the same time (i.e., at retrieval). More specifically, in a cue-based parser, concord and structural constraints are both used in tandem to retrieve a potential attachment site.

Is this a good model of how comprehenders process temporal adverbs? This is the central question of this paper. The cue-based model parsing makes several predictions, which we test in our experiments. Consider the sentences in (3). On our hypothesis about the processing of temporal adverbs, both temporal features and structural features will be used to retrieve an attachment site for the temporal adverbial *last week*. In (3b) there is only one potential attachment site that agrees in all features: the first verb, *taught*. In (3a), however, the syntactically inaccessible verb matches the temporal features of the adverb. This creates the possibility of *similarity-based interference* in the retrieval process (Lewis and Vasishth, 2005). Specifically, the Lewis and Vasishth model predicts *inhibitory interference* in these configurations: the presence of a feature-matched distractor verb *shocked* will slow down retrieval of the target verb *taught*, because in this configuration the feature-matched distractor reduces the amount of activation spread to the target encoding (Jäger et al., 2017). Note that this is only predicted if both tense features and structural features are used as retrieval cues during the processing of the temporal adverb.

(3) a. The musician **taught** the song [that **shocked** everyone] to his new bandmates **last week**.
b. The musician **taught** the song [that will shock everyone] to his new bandmates **last week**.

However, interference can sometimes be *facilitatory* (Jäger et al., 2017). Consider (4). In (4a), no verb agrees with the temporal features of the adverb. Because there is no item in memory that matches the features of the adverb, retrieval will be slow (or may fail), and processing is expected to be difficult. In (4b) however, the distractor verb now matches the tense features of the verb. Thus the target verb *will teach* matches the structural cues, and the distractor verb *shocked* matches the tense cues. This means that the processing of the adverb in (4b) is expected to be faster on average than (4a). This occurs because when there are two verbs that are equally well-matched to the retrieval cues, the overall time to identify a single attachment site is reduced (Jäger et al., 2017; see also Logačev and Vasishth, 2016).

(4) a. The musician will teach the song [that will shock everyone] to his new bandmates **last week**.
b. The musician will teach the song [that **shocked** everyone] to his new bandmates **last week**.

### The Current Study

In the present study, we measured the processing of adverb-verb temporal coherence in sentences as the ones in Table 1 where a structurally accessible attachment site (V1) and an inaccessible attachment site (V2) matched or mismatched the temporal features of the temporal adverb. The resulting four experimental conditions are known as the *mismatch paradigm* (Sturt, 2003).

**Table 1:** Experimental conditions of Experiment 1.

**V1:match,**	(a) The musician *taught* the song that *shocked*
**V2:match**	everyone to his new bandmates last week during
	the dress rehearsal.
**V1:match,**	(b) The musician *taught* the song that *will shock*
**V2:mismatch**	everyone to his new bandmates last week during
	the dress rehearsal.
**V1:mismatch,**	(c) The musician *will teach* the song that *shocked*
**V2:match**	everyone to his new bandmates last week during
	the dress rehearsal.
**V1:mismatch,**	(d) The musician *will teach* the song that *will shock*
**V2:mismatch**	everyone to his new bandmates last week during
	the dress rehearsal.

If comprehenders use only structural information to restrict the retrieval of an attachment site when processing the temporal adverb, then they should be only sensitive to the mis/match in temporal features between the temporal adverb and the structurally accessible verb V1. This should result in longer reading times for the *V1:mismatch* condition compared to the *V1:match* condition. Given that past eye-tracking studies investigating the attachment of temporal adverbs show attachment preferences from early measures on (e.g., Altmann et al., 1998; Van Gompel et al., 2005), we can expect the effect of *V1:match* to be visible from the first-pass to later measures.

Alternatively, if both structural and featural constraints are deployed during the processing of the temporal adverb, the presence of a distractor mis/matching the temporal features of the adverb should affect the retrieval of the licit attachment site. In particular, cue-based parsing models would predict two types of interference: an inhibitory interference effect, with longer reading times for the *V1:match,V2:match* condition when comparing the two *V1:match* conditions, and a facilitatory interference effect, with smaller reading time for the *V1:mismatch,V2:match* condition when comparing the two *V1:mismatch* conditions (Lewis and Vasishth, 2005). A graphic representation^3^ of the four tested conditions, relative retrieval cues and predicted effects from a cue-based perspective is provided in Table 2.

**Table 2:** Graphic representation of the tested conditions and expected effects from a cue-based perspective (Lewis and Vasishth, 2005).

*Condition*	V1	V2	ADVERB	*Prediction*
**(a) V1:match, V2:match**	*Full match*	*Partial match*		
	+PAST	+PAST	+PAST	
	main clause domain	relative clause domain	main clause domain	Inhibitory
**(b) V1:match, V2:mismatch**	*Full match*	*No match*		interference
	+PAST	-PAST	+PAST	(a > b)
	main clause domain	relative clause domain	main clause domain	
**(c) V1:mismatch, V2:match**	*Partial match*	*Partial match*		
	-PAST	+PAST	+PAST	
	main clause domain	relative clause domain	main clause domain	Facilitatory
**(d) V1:mismatch, V2:mismatch**	*Partial match*	*No match*		interference
	-PAST	-PAST	+PAST	(c < d)
	main clause domain	relative clause domain	main clause domain	

*The structural constraint is indicated by the main/relative clause domain. The morphological constraint is represented by +/-PAST. The highlighted cells indicate a match between the retrieval cues provided by the adverb and the target item V1/distractor item V2.*

## Experiment 1

### Methods

#### Participants

Thirty-five undergraduate students from the University of Massachusetts Amherst (31 female, mean age = 20 years, ranging from 18 and 21) participated in this experiment. Participants gave informed consent under an experimental protocol approved by the University of UMass Amherst Institutional Review Board and received course credit for their participation. They were all native speakers of English and had normal or corrected-to-normal vision. Given the absence of past studies addressing our research question, the selection of the sample size was based on past eye-tracking studies^4^ investigating memory retrieval during sentence processing.

#### Materials

A sample of the experimental sentences is provided in Table 1. The experimental material consisted of 24 experimental sentences that were randomly assigned to different lists according to a Latin Square design, so that each subject could see only one version of each item set. Thus, each subject read 6 sentences in each of the four experimental conditions, in addition to 76 grammatical filler sentences (24 of this filler sentences contained a different manipulation that is not reported here). All sentences had the similar length (18–22 words) and the same syntactic structure. The main clause always contained a lexical subject and a ditransitive main verb in either the past tense form, or in the future with *will.* The matrix verb was always followed by two complements of the verb, respectively the direct object (e.g., *the song*) and the indirect object (e.g., *to his new bandmates*), and a temporal adverb followed by some continuations as prepositional phrases or locative adverbs. The embedded relative clause was always attached to the direct object of the main clause and consisted of the complementizer “that” and a past or future verb (e.g., *shocked*/*will shock*) occasionally followed by a direct object (e.g., everyone). The indirect object of the main verb (e.g., *to his new bandmates*) was always a prepositional phrase that followed the relative clause. In order to prevent the prepositional phrase from incorrectly attaching into the relative clause, the verb inside the relative clause was chosen to be syntactically incompatible with this specific prepositional phrase.

In each experimental condition the temporal specification of the deictic temporal adverb (the target word) was held constant; only the temporal features of the two preceding verbs were manipulated. Moreover, to be sure that the two temporal forms were not recognized as always leading to correct (e.g., past) or wrong (e.g., future) verb forms, the experimental material contained the 50% of items with past temporal adverbs (e.g., last month, yesterday) and the other 50% with future adverbs (e.g., next week, tomorrow).

#### Procedure

Eye-movements were recorded using an EYELINK 1000 eye-tracker, with a sampling rate of 1000 Hz. Participants had binocular vision while movements were measured, but only the right eye was tracked. A chin rest bar and a forehead restraint were provided for each participant to minimize head movements. Before the experiment, and whenever necessary during the experiment, the experimenter calibrated the eye-tracker asking participants to fixate nine positions indicated by a black dot, linearly distributed along the central line of the screen. The monitor was positioned 66.3 cm away from the participant, and three characters were subtended by each degree of visual angle. Sentences were presented in 11 point Monaco font via EyeTrack Software^5^. Participants initiated each trial by fixating on a black box on the left side of the screen, specifically where the first word of the sentence would have appeared. Once a fixation in the target region reached a stable value, the entire sentence was displayed, on one single line. After reading, participants ended the presentation of each sentence using one of the buttons of the response pad. Each sentence was followed by a comprehension question concerning the content of the sentence just read (e.g., *Who is going to learn the new song?*). Participants answered by pressing either one of two buttons placed on the response pad corresponding, respectively to the answer on the left (e.g., *The musician*) or on the right (e.g., *The bandmates*) of the screen. The experimental session was preceded by three practice trials to familiarize the participant with the procedure. Testing sessions lasted approximately 1 h, including practice, calibration, break and debriefing.

#### Data Analysis

Sentences were divided into nine regions as shown in (5) separated by the vertical pipe (|). The post-target area was divided in two regions (i.e., post-target, end of the sentence) to divide possible spill-over effects (Just et al., 1982; Mitchell, 1984) in the post-target area due to the experimental manipulation, from general wrap-up effects (Mitchell and Green, 1978; Just and Carpenter, 1980) generally visible at the end of the sentence. Eye-movements were analyzed in three regions of interest: the critical region (e.g., *last week*), the pre-critical region (e.g., *to his new bandmates*) and the post-target region (e.g., *during*).

(5) The musician | taught | the song that | shocked | everyone | to his new bandmates | last week | during | the dress rehearsal.

We report four measures for each region of interest. First, we analyzed *first-pass reading times*, defined as the sum of all fixations on a region of interest before leaving it either to the left or the right. We also analyzed *go-past times* (sometimes called *regression path duration*), defined as the sum of all fixations made once a region of interest has been fixated before moving to the right. Thus, go-past times include time spent re-reading previous regions in addition to the critical region itself. The last reading time measure we report is *total time*, which is the sum of all fixations made on a region of interest, including refixations made after the region has been exited to the right. In addition to these reading time measures, we also report the *probability of regression out*, that is the proportion of times a backward regression was made out of a given region.

Prior to statistical analysis, trials with track loss or blinks in first-pass reading at the critical region were excluded. In this experiment, only one participant was excluded from the analysis because of more than 25% of data loss. The remaining 34 participants (with less than 6% of missing data) reached at least 75% accuracy on the comprehension questions so no participants were excluded due to poor accuracy.

The analysis was carried out fitting linear mixed-effect models to our data, using the R package *lme4* (Bates et al., 2014) and the package *lmerTest* (Kuznetsova et al., 2017) which provides *p*-values in the summary table of each model. The models were built adding *V1:match* as fixed-effects factor, as well as two nested contrasts to test the effect of interference from the illicit distractor V2 both in the V1:match conditions (*c1*) and in the V1:mismatch conditions (*c2*), and crossed random intercepts and random slopes for all fixed-effect parameters both for subject and item grouping factors (Barr et al., 2013). In order to select a parsimonious model which was properly supported by the data, the complexity of the random effect structure of the maximal model was reduced by performing a principal component analysis (PCA; Bates et al., 2015). Only the principal components that were sufficient to cumulatively account for 100% of variance were included in the simplified model. Moreover, the correlation parameters were forced to zero, but only when this further simplification of the model did not significantly decrease the goodness of fit, according to a likelihood ratio test (α_LRT_ = 0.2). The final structure of the best-fitting models is provided in Appendix C.

Our categorical fixed effects predictors were coded using sum-contrast coding. In *V1:match* [V1:match] = 1 and [V1:mismatch] = -1; in *c1*, [V1:match,V2:match] = 1, [V1:match,V2:mismatch] = -1 and [V1:mismatch] = 0; in *c2*, [V1:mismatch,V2:match] = 1, [V1:mismatch,V2:mismatch] = -1 and [V1:match] = 0. For the analysis of the probability of regression measure, logistic mixed-effect models were employed (Jaeger, 2008) using the same coding scheme. The Bonferroni correction was applied to correct the *p*-values for multiple comparisons (von der Malsburg and Angele, 2017). After this correction, a fixed effect was considered significant if its *p*-value was equal or smaller than 0.006.

### Results

**FIGURE 1:**
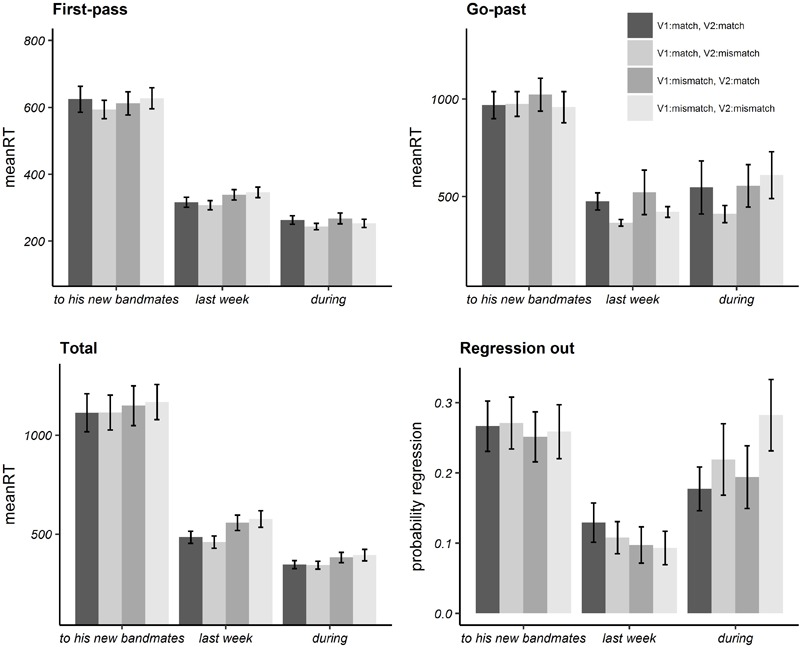
Bar plots of mean reading times in milliseconds in eye-tracking latency measures and mean probabilities of regression out for Experiment 1. Error bars represent standard errors by participant.

Bar plots of mean reading times and probability of regressions in each (pre-target, target, post-target) region are illustrated in Figure 1 while numeric values are given in Appendix A. In Table 3, we report the estimated regression coefficient (Estimate), the standard error (SE) and *t*/Wald’s *z* and *p*-values resulting from the linear mixed-effect model analysis on log-transformed reading times (Baayen and Milin, 2010), for each region.

**Table 3:** Summary of LME analyses of log first-pass, go-past and total time, and probability of regression out for Experiment 1.

	*to his new bandmates*			*last week*			*during*		
First-pass	logRT	*t*	*p*	logRT	*t*	*p*	logRT	*t*	*p*
V1:match	0.002 (0.02)	0.11	0.90	-0.04 (0.01)	-2.21	0.04	-0.004 (0.02)	-0.20	0.85
c1	0.01 (0.03)	-0.28	0.78	0.01 (0.02)	0.51	0.61	-0.027 (0.02)	1.23	0.22
c2	-0.02 (0.03)	-0.80	0.42	-0.01 (0.02)	-0.52	0.60	0.005 (0.02)	0.24	0.81

**Go-past**	**logRT**	***t***	***p***	**logRT**	***t***	***p***	**logRT**	***t***	***p***

V1:match	0.006 (0.02)	0.28	0.78	-0.02 (0.01)	-1.04	0.30	-0.045 (0.03)	-1.45	0.16
c1	-0.009 (0.03)	-0.33	0.74	0.05 (0.03)	1.56	0.12	0.006 (0.04)	0.15	0.88
c2	0.004 (0.03)	0.15	0.89	-0.01 (0.03)	-0.37	0.72	-0.038 (0.04)	-0.87	0.38

**Total**	**logRT**	***t***	***p***	**logRT**	***t***	***p***	**logRT**	***t***	***p***

V1:match	-0.01 (0.02)	-0.55	0.59	-0.08 (0.02)	-3.59	0.001	-0.04 (0.03)	-1.78	0.08
c1	-0.01 (0.03)	-0.41	0.69	0.02 (0.03)	0.59	0.56	0.001 (0.04)	-0.04	0.97
c2	0.02 (0.03)	-0.94	0.35	-0.01 (0.03)	-0.51	0.61	-0.006 (0.04)	-0.24	0.81

**Reg. out**	**prop.**	***z***	***p***	**prop.**	***z***	***p***	**prop.**	***z***	***p***

V1:match	0.02 (0.09)	0.27	0.79	0.10 (0.13)	0.78	0.44	-0.10 (0.13)	-0.82	0.41
c1	-0.02 (0.13)	-0.15	0.88	0.11 (0.18)	0.64	0.52	0.11 (0.18)	-0.59	0.56
c2	-0.03 (0.13)	-0.26	0.80	-0.07 (0.19)	-0.38	0.71	-0.22 (0.17)	-1.24	0.22

*Standard errors are reported in parentheses.*

Analyses on the target region revealed a significant effect of the *V1:match* fixed effect factor, in total reading times, while no significant effects were found in other regions (i.e., pre-target and post-target areas) or measures (i.e., first-pass, go-past, probability of regressions out of a region).

### Discussion

In Experiment 1, reading times significantly increased when the adverb temporal features mismatched the tense features of the main verb of the clause (V1), in late measures (i.e., total time). We found no clear evidence of a significant modulation of the reading times on the adverb as a result of match to the tense features of the embedded verb (V2). However, we note that there is a non-reliable numerical trend that we observed in the go-past measure. At the critical region and spillover region, numerically longer mean go-past times were observed when both verbs matched the temporal features of the adverb. In the spillover region, numerically shorter go-past times were observed when the embedded verb V2 matched the tense features of the adverb. These patterns may be consistent with an inhibitory interference effect and a facilitatory interference effect, respectively (Lewis and Vasishth, 2005, see also Jäger et al., 2017). However, neither of these trends was reliable; we return to these findings in Experiment 2 below.

The main finding from Experiment 1 is that comprehenders are primarily sensitive to the agreement between the temporal features of the adverb and of the matrix verb V1 in incremental sentence processing: reading times were slower when the temporal concord relationship was violated. We interpret these results as evidence that comprehenders retrieve the structurally licit attachment site for the adverb in incremental sentence processing in order to check temporal concord consistency, despite the fact that this verb phrase is linearly more distant than the more recent but more syntactically embedded verb phrase.

Still, the data from Experiment 1 leave open several questions. First, no significant effects were found in early measures (i.e., first-pass) while we observed apparent trends of a V2 match effect in go-past measures and a clear effect of V1 match in total reading times; this leaves open the question of how much interference V2 creates for the attachment of the temporal adverb, at least during sentence re-readings. Second, it is not clear if readers were confident of the appropriate attachment site of the indirect object PP that immediately preceded our temporal adverb, since inflated reading times were found at the PP region. In Experiment 2, we seek to address both of these open questions by replicating and extending our primary finding. We tested the same experimental material of Experiment 1 but added an extra-sentential context preceding each experimental sentence to actively disambiguate the attachment site of the pre-critical region.

## Experiment 2

The goal of Experiment 2 was twofold. First, we wanted to pursue a replication of the primary finding of Experiment 1, namely that readers are primarily sensitive to the V1-adverb match during incremental processing. Second, we decided to extend the paradigm of Experiment 1 adding an extra-sentential context before each sentence, as shown in Table 4.

**Table 4:** Sample of the experimental material of Experiment 2.

**V1:match,**	Tell me more about the musician. To whom did he
**V2:match**	teach the song that shocked everyone?
	(a) The musician *taught* the song that *shocked*
	everyone to his new bandmates last week during
	the dress rehearsal.
**V1:match,**	Tell me more about the musician. To whom did he
**V2:mismatch**	teach the song that will shock everyone?
	(b) The musician *taught* the song that *will shock*
	everyone to his new bandmates last week during
	the dress rehearsal.
**V1:mismatch,**	Tell me more about the musician. To whom will he teach
**V2:match**	the song that shocked everyone?
	(c) The musician *will teach* the song that *shocked*
	everyone to his new bandmates last week during
	the dress rehearsal.
**V1:mismatch,**	Tell me more about the musician. To whom will he teach
**V2:mismatch**	the song that will shock everyone?
	(d) The musician *will teach* the song that *will shock*
	everyone to his new bandmates last week during
	the dress rehearsal.

The goal of this manipulation was to use context to disambiguate the attachment of the prepositional phrase in the pre-critical region, in order to ensure that the effects observed in Experiment 1 were not contaminated by garden-pathing that may have occurred prior to the critical adverb.

We followed Altmann et al. (1998, experiment 2B), who used an interrogative context to guide the attachment of a temporal adverb in sentences such as “She*’ll implement* the plan he *proposed next week*, of course.” In their experiment, the extra-sentential context was manipulated to either focus the temporal adverb *next week* and promote high attachment, e.g., *When will Fiona implement the plan she proposed?* – *She’ll implement the plan* [*she proposed*] *next week, of course*, or to focus a complex noun phrase and therefore favor the low attachment of the temporal adverb, e.g., *Which of the plans she proposed will Fiona implement?* – *She’ll implement the plan* [*she proposed next week*], *of course*.

In our study, we adopted this approach to clarify the attachment of the prepositional phrase *to his new bandmates* to the matrix clause. In our experimental sentences, the prepositional phrase was intended to attach to V1, but it is linearly positioned after the embedded verb V2. We cannot thus exclude that the parser could have been garden-pathed, and temporarily associated this prepositional phrase to V2, although the prepositional phrases were specifically chosen to be incompatible with V2, as outlined above; this could occur either as the result of a structural parsing principle such as *Late Closure* (Frazier, 1979), or as the result of a more general recency preference (MacDonald et al., 1994; Gibson et al., 1996). If the readers were garden-pathed in this fashion—temporarily associating the pre-critical PP to V2—then this could have partially masked the effect of our manipulation or otherwise interfered with the adverb attachment process that immediately follows the PP. This is especially true in rereading measures such as go-past duration: recall that in Experiment 1 we observed a numerical trend toward an interference effect from a structurally inaccessible attachment site. While not reliable, this trend raises the possibility that the V2 distractor matching the temporal cues of the adverb could in fact modulate reading times at the target region, at least in later measures.

To test whether our context manipulation effectively facilitated the interpretation of our experimental sentences, we added 18 filler sentences in which we manipulated the pre-sentential context (see Appendix B for a complete description of this study). The results of this manipulation indicated that the contexts we adopted in Experiment 2 did facilitate the reading of PP and adverb regions, in particular in rereading measures such as go-past and total reading time, thus minimizing any parsing difficulty that may have occurred prior to the critical region.

### Methods

#### Participants

Forty-eight undergraduate students from the UMass Amherst participated in this experiment. They were all native speakers of English and had normal or corrected-to-normal vision, and none had taken part in Experiment 1. Participants gave informed consent under an experimental protocol approved by the UMass Amherst IRB and received course credit for their participation.

#### Materials

The materials in Experiment 2 followed the same design as Experiment 1. However, in Experiment 2 the critical sentences were preceded by a context whose role was to lead the readers expect a PP indirect object of the main verb V1, as shown in Table 4.

Participants were asked to read small dialogues in which there was a character A asking a question (i.e., the pre-sentential context) to a character B. The answer of character B represented the experimental sentence. The context had always the same structure, namely *Tell me more about X. To whom did/will he/she ….?* The first character introduced by the context (e.g., *the musician*) was also the subject/agent of the experimental sentence expressed by a pronoun (i.e., *he* or *she*), while the wh- phrase (i.e., to whom) of the pre-sentential context always referred to the PP of the experimental sentence (e.g., *to his new bandmates*). The experimental sentences provided the answer to a question posed in the extra-sentential context. Comprehension questions targeted information that could have been deduced from various parts of the sentence, aside from the prepositional phrase. Thus, only participants reading the entire target sentence were expected to achieve high comprehension accuracy.

As in Experiment 1, the experimental material consisted of 24 experimental sentences that were randomly assigned to different lists according to a Latin Square design, so that each subject read six sentences in each of the four experimental conditions, in addition to 76 filler sentences (58 simple filler sentences, and 18 filler sentences containing a context manipulation whose description and analysis is reported in Appendix B).

#### Procedure

The same facilities and calibration procedure of Experiment 1 were adopted for the follow-up experiment. However, the procedure for the presentation of the stimuli was different since each trial was composed by a context sentence, an experimental sentence and a comprehension question. Participants initiated each trial by reading the context sentence. After reading the context, participants proceeded to the reading of the experimental sentence using one of the buttons of the response pad. They were asked to fixate on a black box on the left side of the screen, specifically where the first word of the sentence would have appeared. Once a fixation in the target region reached a stable value, the sentence was displayed. After reading, participants ended the presentation of each sentence using one of the buttons of the response pad. Each sentence was followed by a comprehension question concerning the content of the sentence just read. Participants answered by pressing either one of two buttons placed on the response pad corresponding, respectively to the answer on the left or on the right of the screen. The experimental session was preceded by three practice trials to familiarize the participant with the procedure. Testing sessions lasted approximately 1 h, including practice, calibration, break and debriefing.

#### Data Analysis

All features of the analysis were identical to Experiment 1. In this experiment, five participants were excluded from the analysis because of more than 25% of data loss. The remaining 43 participants reached at least 75% accuracy on the comprehension questions; no participants were excluded due to poor accuracy.

### Results

**FIGURE 2:**
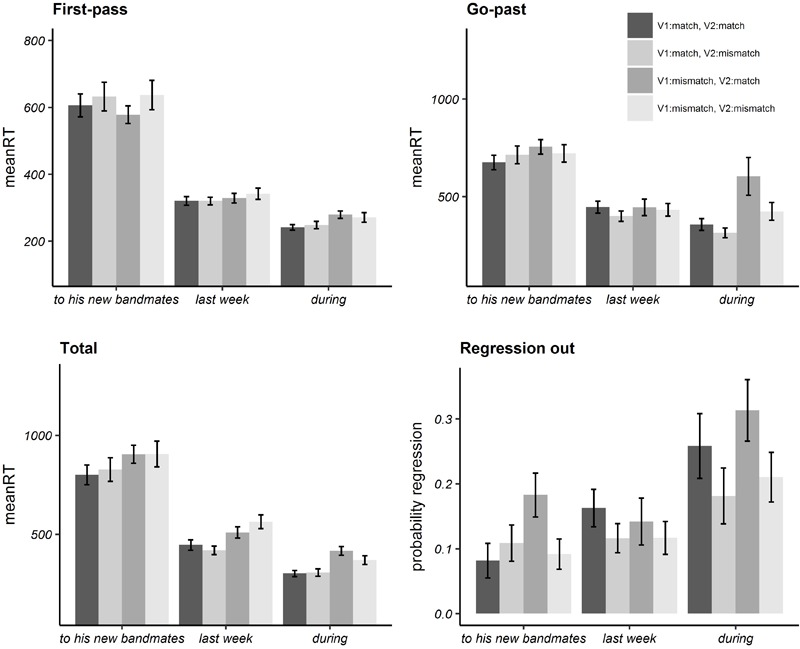
Bar plots of mean reading times in milliseconds in eye-tracking latency measures and mean probabilities of regression out for Experiment 2. Error bars represent standard errors by participant.

Bar plots of mean reading times and probability of regressions in each (pre-target, target, post-target) region are illustrated in Figure 2 while numeric values are given in Appendix A. In Table 5 we report the estimated regression coefficient (Estimate), the standard error (SE), *t*/Wald’s *z* and *p* values resulting from the linear mixed effects model analysis on log-transformed reading times, for each region.

**Table 5:** Summary of LME analyses of log first-pass, go-past and total time, and probability of regression out for Experiment 2.

	*to his new bandmates*			*last week*			*during*		
First-pass	logRT	*t*	*p*	logRT	*t*	*p*	logRT	*t*	*p*
V1:match	0.02 (0.02)	0.94	0.35	-0.03 (0.01)	-0.93	0.35	-0.05 (0.02)	-2.66	0.01
c1	-0.02 (0.02)	-0.77	0.45	-0.003 (0.02)	-0.17	0.87	-0.02 (0.02)	-0.72	0.47
c2	-0.05 (0.02)	-1.86	0.07	-0.02 (0.02)	-1.25	0.21	0.02 (0.02)	1.17	0.25

**Go-past**	**logRT**	***t***	***p***	**logRT**	***t***	***p***	**logRT**	***t***	***p***

V1:match	-0.02 (0.01)	-1.25	0.21	-0.004 (0.02)	-0.23	0.82	-0.12 (0.03)	-4.26	0.0001
c1	-0.03 (0.02)	-1.37	0.17	-0.04 (0.03)	1.30	0.19	0.04 (0.03)	1.13	0.26
c2	0.02 (0.02)	0.76	0.45	-0.01 (0.03)	0.15	0.88	0.06 (0.03)	1.66	0.10

**Total**	**logRT**	***t***	***p***	**logRT**	***t***	***p***	**logRT**	***t***	***p***

V1:match	-0.04 (0.02)	-2.81	0.01	-0.09 (0.02)	-5.20	2.52e-07	-0.12 (0.02)	-5.73	6.18e-07
c1	-0.02 (0.03)	-0.63	0.53	0.02 (0.02)	0.74	0.46	-0.01 (0.03)	-0.25	0.81
c2	0.01 (0.02)	0.66	0.51	-0.04 (0.02)	-1.60	0.11	0.06 (0.03)	2.43	0.02

**Reg. out**	**prop.**	***z***	***p***	**prop.**	***z***	***p***	**prop.**	***z***	***p***

V1:match	-0.25	-1.74	0.08	0.07 (0.13)	0.55	0.58	-0.20 (0.11)	-1.86	0.06
c1	-0.17	-0.88	0.38	0.24 (0.15)	1.58	0.11	0.37 (0.16)	2.25	0.03
c2	0.42	2.65	0.01	0.11 (0.16)	0.71	0.48	0.09 (0.15)	0.60	0.55

Analyses on the target region revealed a significant effect of the *V1:match* fixed effect factor in total reading time, while analyses on the post-target region revealed a significant effect of *V1:match* both in go-past and total reading time.

### Discussion

The results of Experiment 2 present many similarities to, but some differences from Experiment 1. In Experiment 1 we observed inflated reading times on the target adverb when the adverb mismatched the tense features of the main verb of the clause (V1) in late measures (i.e., total time). In Experiment 2 we replicate the same pattern of results on the target region, together with an additional V1 match effect in late measures (i.e., go-past, total time) on the post-target region. Thus like Experiment 1, readers seemed to mainly consider the structurally accessible attachment site for the adverb and the match in features between the verb V1 and the adverb. Overall, the results of Experiment 2 largely confirm the general picture suggested by Experiment 1. Readers were primarily sensitive to the concord between the temporal adverb and the linearly distant, but structurally accessible V1, resulting in a significant effect of *V1:match*. This finding suggests that the parser reliably makes use of structural information to find the right attachment site for the temporal adverb.

Conversely, no statistically significant interference effects were found in the *V1:match* and *V1:mismatch* conditions. According to this analysis, there is not enough evidence to state that the processing of the adverb-verb relation can be modulated by the presence of a structurally illicit but feature matching verb phrase.

## Bayesian Analysis

The clearest result from both Experiments 1 and 2 is that comprehenders are sensitive to a match between the temporal adverb and the (structurally available) verb V1. In Experiment 1, this resulted in a significant reading time slowdown on the critical temporal adverb in total time measures; in Experiment 2, the slowdown was observed in go-past and total times at the spillover, in addition to total times at the critical region. From this, we can confidently conclude that comprehenders incrementally construct a dependency between the temporal adverb and the linearly distant, but structurally accessible, V1.

However, it is less clear whether there is any interference from the features of the grammatically inaccessible V2, as predicted by cue-based parsing models. To evaluate the strength of these findings, we performed a supplementary Bayesian analysis of our data from Experiments 1 and 2. Instead of asking the binary, categorical question “is there interference from V2, or not?” familiar from null hypothesis significance testing (NHST), the Bayesian approach we employ here allows us to ask instead the inherently gradient question “what is the strength of the evidence for interference from V2?” (Nicenboim and Vasishth, 2016).

For this analysis, we used the *rstanarm* package (Stan Development Team, 2016) to fit Bayesian linear mixed effects models to our data. Rather than adopting a parsimonious random effects structure as we did above, we fit ‘maximal’ random effects structures (i.e., varying intercepts and slopes for all fixed effects by subjects and items, along with their correlations; see Barr et al., 2013). This decision was made because maximal random effects structures can be fit without yielding unreasonable results in Bayesian analysis (Nicenboim and Vasishth, 2016). For models of reading times, log-transformed reading times were used as the dependent variable; for models of percent regressions out, we fit a logistic mixed effects model. For each model reported, we fit four Markov chain Monte Carlo (MCMC) chains of 2000 iterations each; unless otherwise noted, the convergence statistic *R-hat* was 1.0 for all parameters estimated. We used the default, weakly informative specification of the prior distributions on model parameters in *rstanarm*, with one exception: we followed Nicenboim and Vasishth (2016) in setting the regularization parameter on the covariance matrix to 2 to promote more conservative estimates of the intercept-slope correlations. In modeling the results in the spillover region of Experiment 2 we performed a prior sensitivity analysis to evaluate whether the choice of prior distribution substantially modified posterior estimates over parameter values (Nicenboim and Vasishth, 2016). We did not find that the choice of prior distribution had a substantial impact on our posterior estimates.

Table 6 summarizes the results as 95% credible intervals over parameter estimates for the models described above. Overall, there is a close alignment between these parameter estimates and those from the planned mixed effects model analysis. For example, in all the regions and measures where we found a statistically significant effect of *V1:match* in the planned linear mixed effects model analysis, we find that the credible intervals in our Bayesian model are quite far from overlapping with 0; we interpret this as evidence that there is clearly an effect of *V1:match* in our data. However, the strength of this Bayesian analysis lies not in making categorical decisions about the presence of absence of an effect, but instead, in quantifying the range of plausible values associated with that effect.

**Table 6:** Summary of Bayesian mixed effects analysis of critical fixed effects.

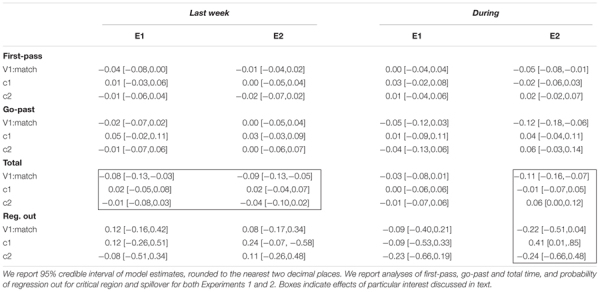

**FIGURE 3:**
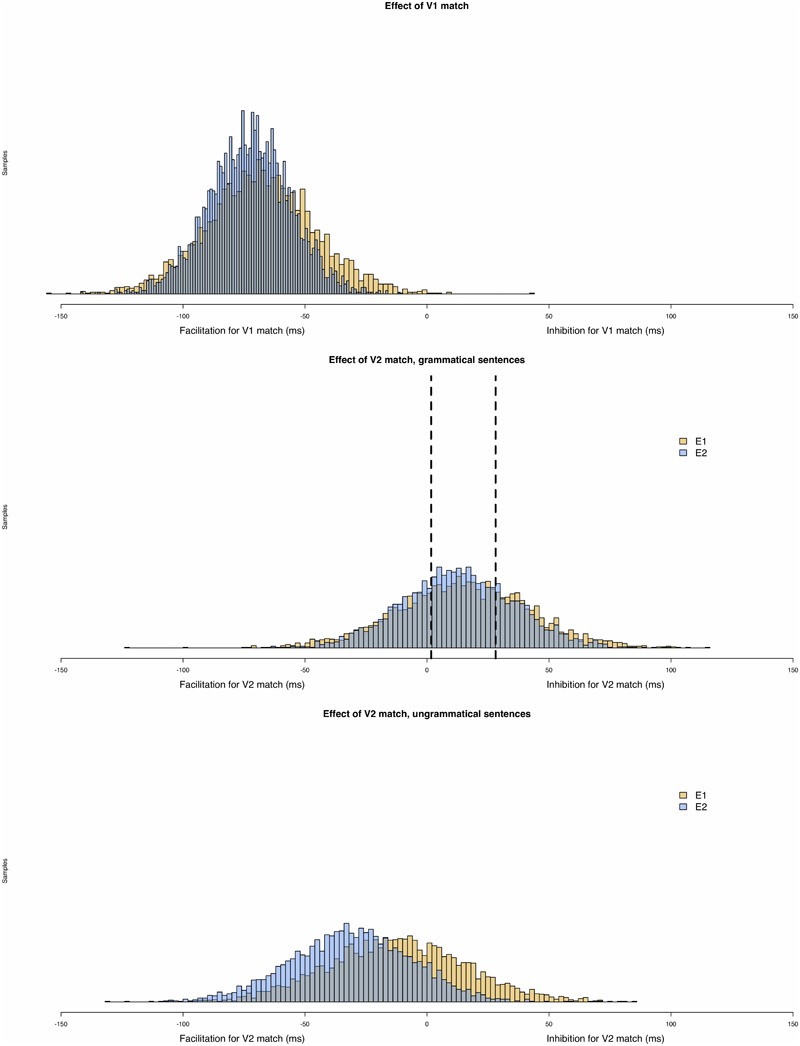
Histogram of the posterior samples for models of total reading times at the critical region in Experiments 1 and 2. The first graph represents the posterior distribution for the V1:match effect, while the second and the third graph represents, respectively the effect of V2:match in *V1:match* conditions (c1) and in *V1:mismatch* conditions (c2).

To aid in this interpretation of our Bayesian analysis, Figure 3 presents a histogram of the posterior samples for models of total reading times at the critical region in both Experiments 1 and 2. As in the NHST analysis, the posterior distribution reveals clear evidence for a V1 match effect, such that reading times were slower when V1 mismatched the adverbial’s temporal features. The picture becomes more interesting when we consider the posterior distribution for the V2 match effect for grammatical sentences (i.e., *V1:match* conditions) in total times at the critical region. On our NHST analysis, this coefficient did not reach statistical significance; correspondingly, the 95% credible intervals in our Bayesian analysis clearly include 0.

However, there are some aspects of our data that may be construed as weak evidence in favor of an inhibitory interference effect of the sort predicted by cue-based parsing models. First, in both experiments, in almost all (13/16) measures the mean effect of the c1 model coefficient is positive, the predicted direction (Jäger et al., 2017). Second, when we compare the posterior distribution for this parameter with the results of Jäger et al. (2017) meta-analysis on interference for subject-verb dependencies, we find that their 95% CI (see dotted lines in Figure 3) aligns with the region of highest density in our parameter estimates. Third, E1 and E2 are in almost complete agreement about the range of plausible values for this parameter in total time measures. Fourth, and finally, we note that there is some evidence that there is a positive value for this coefficient for regressions out in the spillover region of E2. In other words, based on the Bayesian analysis, our data seem to show some evidence for a V2 match effect in grammatical sentences of a magnitude comparable to that observed for inhibitory interference effects for subject-verb dependencies (Jäger et al., 2017). What about a V2 match effect for ungrammatical sentences (i.e., in *V1:mismatch* conditions)? Do we find clear evidence for the predicted facilitatory match effect of a V2 match in this condition? The Bayesian analysis presents less compelling evidence that this is the case. First, there is somewhat less consistency in the direction of this parameter: 10/16 parameter estimates go in the predicted, facilitatory direction. Second, when we do find evidence that the 95% credible interval for this parameter does not include zero (total times in the spillover for E2), the estimated effect goes opposite the predicted direction: we see inhibitory interference (but see Jäger et al., 2017, on a similar pattern observed for reflexive-antecedent dependencies). Third, there is somewhat less cross-experiment consistency in the estimates of this parameter.

It should be noted, however, that this discussion about the presence/absence of a V2 match effect both in grammatical and ungrammatical conditions can be only speculative in this context. The posterior distributions here discussed are too wide and compatible with a wide range of possible results. In order to safely conclude that there is evidence for interference or not, estimates with higher precision than the one presented here (i.e., smaller confidence intervals obtained through higher statistical power) would be needed. In all, our results do not allow us to clearly conclude that there is no effect of V2 match, nor do they clearly allow us to conclude that there is evidence for the (predicted) interference effect of V2 match.

## General Discussion

The main aim of this study was to expand the investigation of concord phenomena in order to understand how linguistic dependencies are processed during sentence comprehension. As we have argued, concord phenomena are useful to study in sentence processing, because the linguistic features marked on one element may provide important cues that can help comprehenders retrieve previously processed linguistic encodings.

In this study, we investigated a different and less typical concord phenomenon, namely the adverb-verb temporal concord relation. We posed some of the questions that are still debated within a cue-based perspective: are all linguistic features candidate cues that guide retrieval? Are all cues given similar weight? Are different cues differently weighted based on the dependency being processed?

We ran two eye-tracking studies in which we tested the processing of deictic temporal adverbs such as *last month* and their dis/agreement in features with two antecedent tensed verbs. Participants read sentences in which a deictic adverb such as *last week* could either agree or disagree in temporal features with a structurally accessible verb (V1) and/or a structurally inaccessible verb (V2) in temporal features. We wished to investigate to what degree the retrieval mechanisms implied during the processing of a temporal adverb are sensitive to structural and/or featural constraints in incremental sentence processing. We expected comprehenders to mainly show sensitivity to the V1-adverb match in the case in which structural information is used to process the adverb-verb relation at long distance. This should have resulted in a main effect *V1:match*. Conversely, we expected reading times to be modulated by the V2-adverb match if both structural and featural information are jointly deployed during the processing of the adverb-verb relation. In this case, two possible interference effect patterns would have been predicted by cue-based parsing models: inhibitory interference in *V1:match* conditions and facilitatory interference in *V1:mismatch* conditions.

Our results reveal two main findings. First, Experiment 1 and Experiment 2 showed that readers were sensitive to the temporal concordance between the adverb and the structurally accessible verb of the main clause V1. When the tense features of V1 mismatched the features of the adverb, longer RTs were observed on the adverb itself in late measures^6^ (i.e., total time) in Experiment 1 and in Experiment 2, and on the word following the adverb (in go-past, total time) in Experiment 2. This pattern of results is further supported by an additional Bayesian analysis which shows that the credible intervals for the effect of *V1:match* do not overlap with 0 exactly in the same regions and measures described above, as well as in the first-pass measure of the post-target region, in Experiment 2.

Second, we did not find unambiguous evidence of interference from the structurally inaccessible verb phrase (V2) in any region or measure of the two experiments, neither in the linear effect model analysis nor in the parameter estimates of the Bayesian analysis. This fails to provide evidence in favor of the claim that both structural and featural cues are used to retrieve a verb to associate the adverb with, as predicted by cue-based parsing models (e.g., Lewis and Vasishth, 2005). However, this failure to find evidence cannot be taken as strong evidence against this view: the low precision of the parameter estimates provided by our Bayesian analysis does not allow us to definitively conclude that an interference effect from V2 is either present or absent. Further studies aiming at increasing the precision of the estimates (e.g., via higher statistical power) are necessary to confidently answer this question. It may be that, the effect size of V2 interference effect (unlike the V1 match effect) is too weak to be detectable in our data without a very large sample size (in the order of hundreds of participants; for a recent discussion on similar topics see Vasishth et al., 2018).

What can be safely concluded from these data is that readers do consider the structurally accessible attachment site (V1) during the processing of the temporal adverb, despite the fact that it is neither the most recent verb, nor the most linearly proximate. In this sense, we may conclude that structural cues guide the processing of the temporal adverb phrase. In what follows we take up some remaining questions from our study, as well as situate our findings on processing temporal adverbs in a broader theoretical context.

### Relating Classical and Cue-Based Approaches to Processing Adverbials

In this paper, we have approached the problem of the attachment and concord between temporal adverbs and verbs from the perspective of cue-based parsing models. It is interesting to consider our theoretical conclusions in light of the broader literature on attachment and concord provided by other psycholinguistic models of sentence parsing.

As reported in the introduction, much of the experimental work on the processing of temporal adverbs has focussed on the processing of syntactically ambiguous sentences such as *John sold the guitar that he found on the beach last week.* There is general consensus in considering the attachment of the adverb to the second verb of the sentence because of general recency effects (e.g., MacDonald et al., 1994) or because of specific parsing principles such as *Late Closure* principle (Frazier, 1979) or *Construal* (Frazier and Clifton, 1996). In light of these earlier claims, our failure to find a strong interference effect from the most recent and linearly closer attachment site V2 may appear surprising. However, this discrepancy is only superficial.

In our study the most recent and linearly closer attachment site does not head the most current thematic domain or argument structure; V1 does. This is because the last phrase which is encountered before attaching the adverb is one of the arguments of the main verb V1 (i.e., the indirect object). Because comprehenders were overwhelmingly sensitive to a V1 match in our data, our findings show that the availability of an attachment site is not gated by simple recency or linear proximate of an attachment site, but it is gated by syntactic structure.

If this conclusion is correct, then one important question that remains is exactly how the parser determines what encodings in memory constitute structurally available attachment sites for the adverb (see Kush, 2013, for an extended discussion of the theoretical issues). We are not in a position to offer a definitive answer to this question, but we see this as an exciting area of future research. One possible implementation of this idea is to borrow Construal’s claim that the current thematic domain is what defines which attachment sites are syntactically available for the temporal adverb. This could perhaps be implemented as a retrieval cue that matches material in a current thematic domain. Such a model would integrate Construal’s claim about what constitutes a licit attachment site (the current thematic/syntactic domain), with the cue-based retrieval mechanism for forming attachment relations in a model such as ACT-R (Lewis and Vasishth, 2005). This possibility is highly speculative at present, but this perspective could provide a useful avenue for further addressing the interplay between cue-based and structured processing models.

There is one important limitation of our study that bears further discussion. The type of sentence structure we adopted in this study allowed us to disentangle simply recency from structural factors in the investigation of adverb attachment. However, an open question is whether the same sentence structure was ideal to test interference effects from the illicit distractor. In the framework of a model such as the one proposed by Lewis and Vasishth (2005), the processing of the indirect object of the main verb of the sentence (i.e., to his new bandmates) may reactivate and strengthen the encoding of V1 or its associated verb phrase (Lewis et al., 2006; Vasishth and Lewis, 2006). This is because this constituent is still a dependent of V1, even if it is not directly involved in the long-distance temporal concord dependency. If this line of reasoning is correct, the increased activation of V1 (or its associated verb phrase) could have diminished the strength of any interference effect from V2, because V2 would be relatively less active. This line of reasoning is consistent with the presence of some residual/weak interference in the Bayesian analysis. This is a general design issue for studies looking at interference effects that bears closer scrutiny, since models such as Lewis and Vasishth’s predict that covert reactivation of constituents boosts their activation in working memory. For example, the retrieval of the licit antecedent of a reflexive pronoun has been investigated in sentences such as “[*The surgeon* [*who treated Jennifer*] *had pricked himself*]…” (example provided by Jäger et al., 2017 in their meta-analysis, taken from Sturt (2003)). Based on the reactivation account, the VP (*had pricked*) of the sentence can have, in principle, re-activated its argument (i.e., the licit NP *the surgeon*), thus lowering the activation of the illicit distractor (*Jennifer*) before encountering the reflexive pronoun. Dillon et al. (2013) explored such a ‘reactivation-based’ account of the diminished reflexive intrusion effect but argued using computational simulations that the reactivation boost was not sufficient to predict the observed lack of intrusion effects. Dillon et al.’s (2013) results suggest that the reactivation of the target can diminish interference effects from the distractor, although in those simulations it did not totally eliminate those interference effects. It is difficult to compare the results of those simulations too directly to our present materials, although they do provide a proof of concept that the concern about target reactivation is well-placed, although in those simulations it did seem that interference from the distractor was still predicted. In general, it is difficult to reason about the impact of this reactivation process without the aid of an implemented computational model, and it is beyond the scope of the present project to simulate this process.

### Interference Across Dependencies

We have highlighted the fact that in our data, comprehenders were clearly sensitive to a V1 match. We have interpreted this as evidence for the immediate application of structural constraints in the selection of an attachment site.

One important motivation for our study was to broaden the empirical base on which cue-based models are founded and include a novel linguistic relation: temporal adverb – verb dependencies. How does the processing of these adverbs compare to other dependencies that have been studied in this literature? Recent evidence has shown that the presence and the direction of the interference effect, both in grammatical and ungrammatical conditions, is not consistent across dependencies (Jäger et al., 2017). With respect to temporal adverbs, our Bayesian analysis has shown that the most compelling evidence for interference from an illicit attachment site seems to arise only in the grammatical conditions, in the form of inhibitory interference. There was not clear evidence for facilitatory interference in the ungrammatical conditions. This pattern should be supported by stronger experimental evidence in order to be extensively discussed. However, if we speculatively take this as the interference profile for temporal adverbs, then this sets them apart from both reflexive and subject-verb agreement dependencies. These dependencies show, respectively, small or strong evidence for interference (facilitatory in the case of subject-verb agreement, inhibitory in case of reflexives), but only in the ungrammatical conditions. Temporal adverbs seem more similar to subject-verb dependencies, which show an inhibitory effect in the grammatical conditions (Jäger et al., 2017). A more complete comparison between subject-verb attachment and verb-adverb attachment is not possible at present, however, because interference on subject-verb attachment has not been tested in ungrammatical conditions. Thus, further research is needed to show whether this dependency fully matches the results we found for adverb attachment, or whether the subject-verb dependency would show a reliable facilitatory effect in the ungrammatical conditions, as predicted by Lewis and Vasishth’s (2005) model.

Our finding that structural constraints are immediately applied in the processing of temporal adverbs are also partially in line with some recent proposals that draw a link between the priority that structural constraints can have during retrieval and the predictability of the dependency under computation. In particular, Parker and Phillips (2017) proposed that the relative unpredictability of reflexives may be the source of their structure sensitivity found during the processing of the reflexive dependency, especially as compared to the processing of subject-verb agreement dependencies. Similarly, we could extend this proposal to the processing of temporal adverb attachment. It may be that the strong role played by structural constraints during the processing of the adverb can be related to the unpredictability and optionality of the adverb constituent.

However, reflexives and temporal adverbs are unpredictable in somewhat different ways that complicate this comparison. While a reflexive and its linguistic features cannot be predicted, *per se*, the structural position of a reflexive *can* be predicted: this is typically the direct object position of a verb phrase, and this position can be easily predicted, at least for verbs with a specific subcategorization frame (e.g., transitive verbs). Conversely, the attachment site of the temporal adverb is not predictable and needs to be built “from scratch” once the adverb is encountered. In other words, for reflexives attachment processes may be selectively facilitated by predictive processing, leaving only concord and binding processes to be resolved through memory retrieval. But in the case of temporal adverbs, neither attachment nor concord processes are likely to benefit from predictive processes, as we have emphasized. It is currently hard to imagine whether this more subtle difference plays a role in the pattern of findings provided by the current experimental literature. Further research is primarily needed in order to better assess to which extent the attachment of temporal adverbs is prone to interference and to confirm or disconfirm the pattern of results provided by the current study.

Another potential—but at present speculative—explanation is that the attachment of a temporal adverb fails to show the complete predicted pattern of interference because of the specific sentential context we adopted in our study. In this study we tested adverb attachment in isolated sentences or in presence of a context whose role was to clarify the interpretation of the pre-target constituent. By varying syntactic configurations, or by varying the amount of distractor to target match in the items, it may still be possible to observe clearer inhibitory and facilitatory interference effects during adverb attachment by adopting different experimental designs that raise the salience of the distractor verb. This possibility seems particularly plausible if we consider experimental findings on the processing of reflexives in detail. Some studies on the processing of reflexives failed to find interference effects from illicit distractors (e.g., Nicol and Swinney, 1989; Sturt, 2003; Xiang et al., 2009; Dillon et al., 2013; Cunnings and Sturt, 2014), though more recent studies have shown that the prominence of the illicit distractor or the degree of feature match of the licit antecedent can increase the strength of interference effects during the processing of reflexives (Patil et al., 2016; Parker and Phillips, 2017; see also Sloggett, 2017). In other words, we cannot exclude that other factors, including non-syntactic ones, may increase the sensitivity to syntactically illicit attachment sites during the processing of a temporal adverb. Further research is needed to identify which factors may boost interference effects during adverb attachment.

## Conclusion

The central question of the current study was whether comprehenders use syntactic positional information and/or (temporal) featural information to process the adverb-verb temporal concord relation at long distance during sentence processing. In two eye-tracking studies, we found consistent evidence that comprehenders use structural information to determine the attachment site for the temporal adverb and process the concord relation. Further research is needed to establish whether other, non-structural factors may also play a role during the processing of the adverb-verb temporal relation.

## Ethics Statement

The protocol (nr. 2015–2661) was approved by the UMass Amherst Institutional Review Board. All participants gave written informed consent.

## Author Contributions

NB wrote the original draft and was responsible for the conception and the design of the work, as well as for the acquisition, analysis and interpretation of the data. FV contributed to the original draft and to the interpretation of the data. BD contributed to the original draft, as well as to the design, to the analysis and interpretation of the data.

## Conflict of Interest Statement

The authors declare that the research was conducted in the absence of any commercial or financial relationships that could be construed as a potential conflict of interest.

## Footnotes

1 Other theoretical approaches posit a different syntactic position for adverbs, such as the specifier in a dedicated functional projection for tense (e.g., Alexiadou, 1997, 2000; Cinque, 1999, 2004). On this view, the temporal adverb is located in the specifier of the TP, and the tense in the head T. This local syntactic configuration then is what permits the two constituents to concord in temporal features and build up a coherent temporal interpretation of the event. However, the differences between these syntactic accounts are not crucial for the present study.

2 Deictic temporal adverbs belong to a specific category of temporal adverbs that need to be anchored to the time of utterance (i.e., yesterday defines the 24-hour time interval preceding the time of utterance “now”). They differ from other adverbs such as clock-calendar adverbs (e.g., at noon, at 5 PM) or dependent adverbs (e.g., previously, afterward) that may be, or never are, anchored to the time of utterance (Smith, 1978, 1981). Deictic temporal adverbs and clock-calendar adverbs do not lead to similar temporal inconsistencies (e.g., I left/^∗^will leave yesterday; I left/will leave at 5 PM).

3 For the realization of this table we took inspiration from Figure 1 in Jäger et al. (2017)

4 The search was conducted in the database Web of Science by using the following keywords “memory AND retrieval AND eye AND movements AND sentence.” The list of papers published in the last 10 years was then integrated with the list of eye-tracking studies reported in the recent review by Jäger et al. (2017).

5 http://blogs.umass.edu/eyelab/software/

6 Previous eye-tracking studies have shown significant effects of adverb attachment/concord from early measures on (e.g., Altmann et al., 1998; Van Gompel et al., 2005). One possible explanation of this discrepancy could be methodological. We adjusted alpha for multiple comparisons while the aforementioned studies did not. Indeed, without the alpha adjustment our data would have also given a significant effect of *V1:match* in first-pass (see first-pass of the target region in Experiment 1, first-pass of the post-target region in Experiment 2).
