# Supplementary material for: Attachment and Concord of Temporal Adverbs: Evidence From Eye Movements
Source: Front Psychol. 2019 May 31;10:983. doi: 10.3389/fpsyg.2019.00983 (PMC6555130; doi:10.3389/fpsyg.2019.00983)
Supplement: Supplementary file 1 [file Data_Sheet_1.docx]

**Appendix A**

Raw reading times for Experiment 1 are shown in Table A1, while raw reading times for Experiment 2 are shown in Table A2.

**Table A1.** Mean first-pass, go-past, total reading times in milliseconds and probabilities of regression out for Experiment 1. Standard errors are reported in brackets.

|  | *to his new bandmates* | *last week* | *during* |
| --- | --- | --- | --- |
| **First-pass** |  |  |  |
| V1:match, V2:match | 624.2 (38.9) | 316.5 (15.1) | 263.1 (12.4) |
| V1:match, V2:mismatch | 593.8 (27.4) | 307.3 (13.9) | 243.6 (9.4) |
| V1:mismatch, V2:match | 612 (34.6) | 338.5 (15.4) | 267.9 (16.2) |
| V1:mismatch, V2:mismatch | 627.3 (31.4) | 345.6 (15.9) | 253.3 (12.5) |
| **Go-past** |  |  |  |
| V1:match, V2:match | 968.8 (69.2) | 475 (43.7) | 547 (136) |
| V1:match, V2:mismatch | 974.4 (63.1) | 365.2 (17.1) | 410.2 (44.6) |
| V1:mismatch, V2:match | 1022.3 (83.9) | 521.3 (114.6) | 554.8 (109) |
| V1:mismatch, V2:mismatch | 957.7 (80.1) | 421.2 (27.5) | 609.5 (120.3) |
| **Total time** |  |  |  |
| V1:match, V2:match | 1113.3 (96) | 484.1 (30.4) | 346 (20.2) |
| V1:match, V2:mismatch | 1114.3 (88.6) | 459.5 (30.7) | 342.9 (19.2) |
| V1:mismatch, V2:match | 1148.6 (99.9) | 557.1 (38.5) | 381.9 (26.2) |
| V1:mismatch, V2:mismatch | 1166.8 (88.9) | 575.7 (41.8) | 393.7 (28.7) |
| **Pr. regression out** |  |  |  |
| V1:match, V2:match | 0.27 (0.04) | 0.13 (0.03) | 0.18 (0.03) |
| V1:match, V2:mismatch | 0.27 (0.04) | 0.11 (0.02) | 0.22 (0.05) |
| V1:mismatch, V2:match | 0.25 (0.04) | 0.1 (0.03) | 0.19 (0.04) |
| V1:mismatch, V2:mismatch | 0.26 (0.04) | 0.09 (0.02) | 0.28 (0.05) |

**Table A2.** Mean first-pass, go-past, total reading times and probabilities of regression out for Experiment 2. Standard errors are reported in brackets.

|  | *to his new bandmates* | *last week* | *during* |
| --- | --- | --- | --- |
| **First-pass** |  |  |  |
| V1:match, V2:match | 606 (34.2) | 320.2 (13.1) | 241.5 (8.4) |
| V1:match, V2:mismatch | 632.3 (42.7) | 320.2 (11.4) | 248.3 (10.7) |
| V1:mismatch, V2:match | 577.4 (26.1) | 328.6 (14.3) | 279 (11) |
| V1:mismatch, V2:mismatch | 637 (43.9) | 341.8 (16.9) | 270.7 (14.4) |
| **Go-past** |  |  |  |
| V1:match, V2:match | 675.1 (36.8) | 446 (30.8) | 357 (30.1) |
| V1:match, V2:mismatch | 713.9 (45.7) | 399.6 (26.2) | 314.6 (24.6) |
| V1:mismatch, V2:match | 755 (36.8) | 445.1 (42) | 604 (96.3) |
| V1:mismatch, V2:mismatch | 721.5 (44.9) | 432.5 (32.2) | 424.2 (44.9) |
| **Total time** |  |  |  |
| V1:match, V2:match | 800 (49.6) | 445.8 (25.9) | 301.8 (16.2) |
| V1:match, V2:mismatch | 826.8 (59.6) | 418.7 (21.3) | 306.8 (18.8) |
| V1:mismatch, V2:match | 904.5 (45.2) | 509.1 (28.6) | 416.2 (22.2) |
| V1:mismatch, V2:mismatch | 905.9 (64.9) | 563 (34.7) | 369.4 (22) |
| **Pr. regression out** |  |  |  |
| V1:match, V2:match | 0.08 (0.03) | 0.16 (0.03) | 0.26 (0.05) |
| V1:match, V2:mismatch | 0.11 (0.03) | 0.12 (0.02) | 0.18 (0.04) |
| V1:mismatch, V2:match | 0.18 (0.03) | 0.14 (0.04) | 0.31 (0.05) |
| V1:mismatch, V2:mismatch | 0.09 (0.02) | 0.12 (0.03) | 0.21 (0.04) |

**Appendix B**

**Context manipulation in Experiment 2**

To test whether the manipulation of the pre-sentential context played a role of facilitation while reading the prepositional phrase (PP) and the following word, 18 filler sentences with three different manipulations were added in Experiment 2. We report the three manipulations of the pre-sentential context in (4).

(4) a) Tell me more about the student. To which teacher did he dedicate the prize that he won?

He dedicated the prize that he won to the teacher that supported him during high school.

b) Tell me more about the student. What did he do?

He dedicated the prize that he won to the teacher that supported him during high school.

c) Tell me more about the student. What did he do?

He dedicated the prize to the teacher that supported him during high school.

The *PP-focused context* condition in (4.a) contained the same type of context that was adopted for the experimental sentences. The context-question (e.g. *To which teacher did he…?*) has been built to focus the PP (e.g. *to the teacher that…*) in the experimental sentence.

The *plain context* condition in (4.b) contained a context-question (e.g. *What did he do?*) that makes the entire sentence the focus-answer to the question (e.g. *He dedicated…*). If the context in (4.a) allows the parser to attach more easily the PP in the sentence and interpret it as an argument of V1, instead of V2, we expect smaller reading times at the PP region and at the following one in (4.a) compared to (4.b).

Moreover, we added the *plain context (no relative clause)* condition in (4.c) to test whether the difficulty in processing the PP was only driven by the presence of an embedded relative clause. Similarly to the condition in (4.b), the condition in (4.c) contained a context-question that gives a broad focus and deals with the entire experimental sentence. However, the experimental sentence in (4.c) differs from the other two conditions because it does not contain the embedded relative clause (e.g. *that he won*) before the prepositional phrase. A different set of predictions follows from the assumption that the processing difficulties are driven by the presence of the relative clause: as the context is equal, we expect longer reading times for the *plain context* condition in (4.b) compared to the *plain context (no relative clause)* condition in (4.c).

**Method**

**Participants and procedure.** The same participants, facilities, calibration procedure and task of Experiment 2 were adopted since this study was included in Experiment 2.

**Materials.** Each of the 18 sentences was manipulated in three different conditions and were divided in the different experimental lists following a Latin-square design. The three conditions differed either in the type of context (*PP-context* vs *plain context, plain context (no relative clause))* or in the presence of the relative clause in the target sentence (*PP-context, plain context* vs *plain context (no relative clause))*.

Sentences were built as similar as possible to the experimental sentences of Experiment 2: they contained a main subject noun phrase, the verb, the object noun phrase, an embedded relative clause, a prepositional phrase and a relative clause starting with the complementizer “that”. Half of the sentences contained past verb forms, while the other half contained future verb forms. As for the other trials of Experiment 2, comprehension questions dealt with information that could have been deduced reading other parts of the sentence, besides the prepositional phrase of the experimental sentence. In this way, only participants reading the entire sentence also reached high accuracy rates.

**Data analysis.** Sentences were divided into 7 regions (e.g. He | dedicated | the prize | that he won | to the teacher | that | supported him during high school) but only the relevant (target and post-target) region were analysed, namely the target region (e.g. to the teacher) and the post-target region (e.g. that).

Prior to statistical analysis, trials with track loss or blinks in first-pass reading at the critical region were excluded. In this experiment, 5 participants were discarded because of high proportion (>25%) of missing data so analyses were run on 43 participants, who all reached at least 75% accuracy on the comprehension questions.

The analysis was carried out ﬁtting linear mixed-effect models to our data. The models were built considering the fixed-effect factor *condition* with three-levels (namely pp-focused, sentence-focused, no-relative fixed-effects factor), and considering random intercepts and random slopes for the fixed-effect parameter both for subject and item grouping factors (Barr et al. 2013). The same criteria adopted in the two previous experiments (i.e. model selection criterion using a parsimonious approach, Bonferroni correction for multiple comparisons) were applied on these data.

The comparison between the sentence-focused condition (4.b) and either the PP-focused condition (4.a) or the no-relative clause condition (4.c) was carried out by setting the sentence-focused condition as the reference level of the intercept in all models. We report four measures for each region of interest: first pass reading time, go-past time, total reading time and probability of regressions out.

**Results**

Bar plots of mean reading times and probability of regressions in the target and post-target region are illustrated in Figure 4 while numeric values are given in Table B1. In Table B2, we report the estimated regression coefficient (Estimate), the standard error (SE) and p values resulting from the linear mixed effects model analysis on log-transformed reading times, for each region.

Data at the target (PP) region and at the post-target (complementizer) region both showed significantly faster reading times for the PP-context condition compared to the sentence-focused condition in go-past and total time. Smaller probabilities of regression out of the target and the post-target region were also reported for the PP-context condition compared to the sentence-focused condition.

Conversely, no significant differences were found between the reading times of the sentence-focused condition and the no-relative condition at the target region and post-target region.


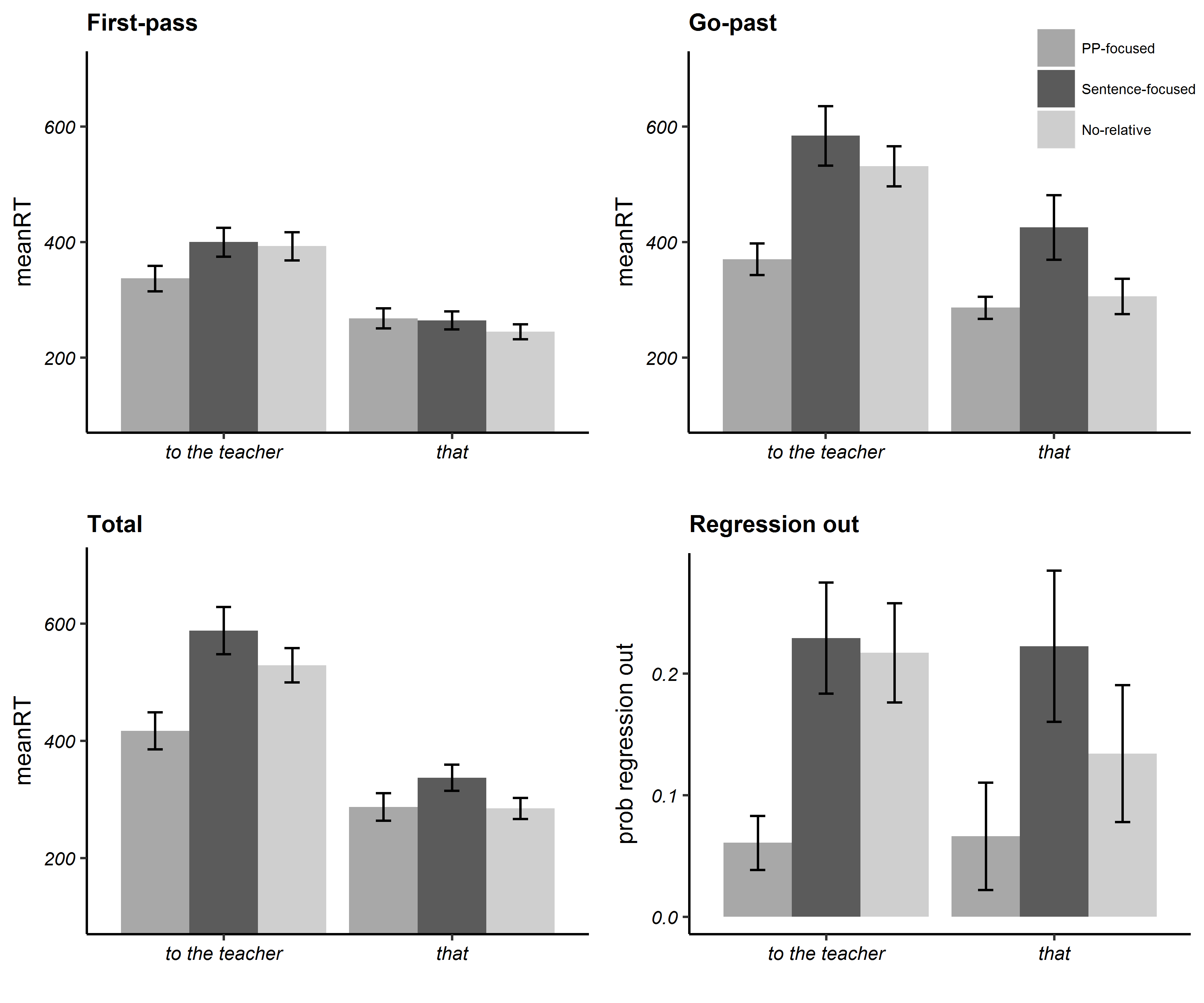


Figure 4. Bar plots of mean reading times in milliseconds in eye-tracking latency measures and mean probabilities of regression out for the context manipulation of the filler sentences in Experiment 2. Error bars represent standard errors by participant.

**Table B1.** Mean first-pass, go-past, total reading times and probabilities of regression out for the context manipulation of the filler sentences in Experiment 2. Standard errors are reported in brackets.

|  | *to the teacher* | *that* |
| --- | --- | --- |
| **First-pass** |  |  |
| Sentence-focused | 400 (25) | 264 (15) |
| PP-focused | 337 (22) | 268 (17) |
| No-relative clause | 393 (24) | 245 (13) |
| **Go-past** |  |  |
| Sentence-focused | 584 (51) | 425 (56) |
| PP-focused | 370 (27) | 286 (19) |
| No-relative clause | 531 (35) | 306 (31) |
| **Total time** |  |  |
| Sentence-focused | 588 (40) | 337 (22) |
| PP-focused | 417 (32) | 287 (23) |
| No-relative clause | 529 (29) | 285 (18) |
| **Pr. regression out** |  |  |
| Sentence-focused | 0.23 (0.05) | 0.22 (0.06) |
| PP-focused | 0.06 (0.02) | 0.07 (0.04) |
| No-relative clause | 0.22 (0.04) | 0.13 (0.06) |

**Table B2.** Summary of LME analyses of log first-pass, go-past and total time, and probability of regression out for the context manipulation of the filler sentences of Experiment 2. Standard errors are reported in parentheses.

|  | *to the teacher* | | | *that* | | |
| --- | --- | --- | --- | --- | --- | --- |
| **First-pass** | logRT | *t* | *p* | logRT | *t* | *p* |
| Sentence-focused | 5.86 (0.06) | **-** | - | 5.50 (0.05) | **-** | - |
| PP-focused | -0.13 (0.08) | -1.68 | 0.11 | -0.03 (0.05) | -0.59 | 0.57 |
| No-relative clause | 0.01 (0.07) | 0.19 | 0.85 | -0.03 (0.05) | -0.56 | 0.58 |
| **Go-past** | logRT | *t* | *p* | logRT | *t* | *p* |
| Sentence-focused | 6.15 (0.07) | **-** | - | 5.79 (0.05) | **-** | - |
| PP-focused | -0.36 (0.08) | -4.75 | 0.0001 | -0.24 (0.08) | -3.19 | 0.005 |
| No-relative clause | -0.03 (0.10) | -0.32 | 0.76 | -0.19 (0.07) | -2.54 | 0.02 |
| **Total time** | logRT | *t* | *p* | logRT | *t* | *p* |
| Sentence-focused | 6.22 (0.07) | **-** | - | 5.71 (0.06) | **-** | - |
| PP-focused | -0.33 (0.08) | -4.35 | 0.0004 | -0.21 (0.06) | -3.28 | 0.004 |
| No-relative clause | -0.07 (0.10) | -0.72 | 0.48 | -0.14 (0.07) | -2.05 | 0.05 |
| **Pr. regression out** | prop. | *z* | *p* | prop. | *z* | *p* |
| Sentence-focused | -1.38 (0.25) | - | - | -1.50 (0.31) | - | - |
| PP-focused | -1.54 (0.38) | -4.03 | 5.71e-05 | -1.31 (0.45) | -2.93 | 0.003 |
| No-relative clause | -0.25 (0.42) | -0.59 | 0.56 | -0.80 (0.38) | -2.12 | 0.03 |

**Discussion**

In this study, we manipulated two variables: on one hand we manipulated the pre-sentential context which can either focus one specific phrase of the experimental sentence (i.e. PP-focused condition) or the entire sentence (i.e. plain context condition), on the other hand we manipulated the complexity of the experimental sentence either considering the whole sentence containing an embedded relative clause (i.e. plain context condition) or considering sentences in which the embedded clause was removed (i.e. plain context (no relative clause) condition).

In the context manipulation, we predicted the PP-focused condition to facilitate the reading and the interpretation of the target word (i.e. the prepositional phrase) and the following one compared to the condition in which the context does not put in focus any specific phrase, in particular in rereading measures. Our findings support our prediction since we found significantly smaller probabilities of regression out of the target and post-target region, as well as faster reading times both at the target and pre-target region in go-past and total reading times. In other words, comprehenders tend to spend more time in rereading the sentence when the context does not provide any specific focus to any specific referent.

Further confirmation of these findings comes from the sentence manipulation we designed within the same study. In fact, one possible explanation about the longer rereading times at the PP region could be the presence of an embedded relative clause, which makes the sentence and the indirect object of the main verb more complex to process. Data coming from the sentence manipulation showed that the presence or absence of an embedded relative clause does not affect reading times at the PP region and the following one, in any measures, although some numerical differences are visible on the word following the target, late in processing (i.e. go-past and total time).

In sum, data coming from this set of sentences give positive evidence with respect to the role of facilitation played by a “focused” context during the interpretation of a prepositional phrase, compared to a plain context. This is crucial for our interpretation of Experiment 2, as it suggests that adopting a pre-sentential context significantly facilitated the attachment and the interpretation of the (pre-target) prepositional phrase and the following words. In other words, any potential pre-critical garden path was minimized by our contexts, at least in rereading measures (i.e. go-past, total). In addition, these findings provide a conceptual replication of Altmann et al.’s findings (1998) showing that the attachment of adjunct phrases in a potentially ambiguous sentence environment can be overridden by a specific (focusing) context, at least during sentence rereading.

**Appendix C**

Final structure of the linear mixed effect models for each experiment, measure and region of interest. The analysis was conducted using the lme4 package, in R studio software. The most complex model ( log (value) ~ V1:match + c1 + c2 + (1 + V1:match + c1 + c2 | subj) + (1 + V1:match + c1 + c2 | item) ) was simplified by adopting the Principal Component analysis developed by Bates et al. 2015.

**1. Experiment 1**

**1.1. First pass**

**1.1.1. Pre-target**

m = log(value) ~ V1:match + c1 + c2 + (1 | subj) + (1 | item)

**1.1.2. Target**

m = log(value) ~ V1:match + c1 + c2 + ((1 | subj) + (0 + V1:match | subj)) + ((1 | item) + (0 + V1:match | item))

**1.1.3. Post-target**

m = log(value) ~ V1:match + c1 + c2 + (1 + V1:match | subj) + (1 + V1:match | item)

**1.2. Go-past**

**1.2.1. Pre-target**

m = log(value) ~ V1:match + c1 + c2 + (1 | subj) + ((1 | item) + (0 + V1:match | item))

**1.2.2. Target**

m = log(value) ~ V1:match + c1 + c2 + (1 | subj) + (1 | item)

**1.2.3. Post-target**

m = log(value) ~ V1:match + c1 + c2 + (1 + V1:match | subj) + (1 | item)

**1.3. Total**

**1.3.1. Pre-target**

m = log(value) ~ V1:match + c1 + c2 + ((1 | subj) + (0 + V1:match | subj)) + ((1 | item) + (0 + V1:match | item) + (0 + c1 | item))

**1.3.2. Target**

m = log(value) ~ V1:match + c1 + c2 + ((1 | subj) + (0 + V1:match | subj) + (0 + c1 | subj)) + (1 | item)

**1.3.3. Post-target**

m = log(value) ~ V1:match + c1 + c2 + (1 | subj) + (1 | item)

**1.4. Probability regression out**

**1.4.1. Pre-target**

m = value ~ V1:match + c1 + c2 + (1 | subj) + (1 | item)

**1.4.2. Target**

m = value ~ V1:match + c1 + c2 + (1 | subj) + (1 | item)

**1.4.3. Post-target**

m = value ~ V1:match + c1 + c2 + (1 | subj) + (1 | item)

**2. Experiment 2**

**2.1. First pass**

**2.1.1. Pre-target**

m = log(value) ~ V1:match + c1 + c2 + ((1 | subj) + (0 + V1:match | subj) + (0 + c1 | subj) + (0 + c2 | subj)) + (1 | item)

**2.1.2. Target**

m = log(value) ~ V1:match + c1 + c2 + (1 | subj) + (1 | item)

**2.1.3. Post-target**

m = log(value) ~ V1:match + c1 + c2 + (1 | subj) + ((1 | item) + (0 + V1:match | item))

**2.2. Go-past**

**2.2.1. Pre-target**

m = log(value) ~ V1:match + c1 + c2 + ((1 | subj) + (0 + V1:match | subj)) + (1 | item)

**2.2.2. Target**

m = log(value) ~ V1:match + c1 + c2 + (1 | subj) + ((1 | item) + (0 + V1:match | item))

**2.2.3. Post-target**

m = log(value) ~ V1:match + c1 + c2 + (1 | subj) + (1 + V1:match | item)

**2.3. Total**

**2.3.1. Pre-target**

m = log(value) ~ V1:match + c1 + c2 + ((1 | subj) + (0 + V1:match | subj) + (0 + c1 | subj)) + (1 | item)

**2.3.2. Target**

m = log(value) ~ V1:match + c1 + c2 + (1 | subj) + (1 | item)

**2.3.3. Post-target**

m = log(value) ~ V1:match + c1 + c2 + ((1 | subj) + (0 + V1:match | subj)) + (1 | item)

**2.4. Probability regression out**

**2.4.1. Pre-target**

m = value ~ V1:match + c1 + c2 + (1 + V1:match + c1 + c2 || subj) + (1 | item)

**2.4.2. Target**

m = value ~ V1:match + c1 + c2 + (1 | subj) + (1 + V1:match || item)

**2.4.3. Post-target**

m = value ~ V1:match + c1 + c2 + (1 | subj) + (1 + V1:match || item)
